# Supplementary material for: Dihydrotanshinone I Enhances Cell Adhesion and Inhibits Cell Migration in Osteosarcoma U−2 OS Cells through CD44 and Chemokine Signaling
Source: Molecules. 2022 Jun 9;27(12):3714. doi: 10.3390/molecules27123714 (PMC9231138; doi:10.3390/molecules27123714)
Supplement: Supplementary file 1 [file molecules-27-03714-s001.zip › molecules-1734415-supplementary.pdf]

Dihydrotanshinone I enhances cell adhesion and inhibits cell migration in osteosarcoma U-2 OS cell through CD44 and chemokine signaling

Lanyan Fan[1, 2], Chen Peng[2], Xiaoping Zhu[1, 2], Yawen Liang[2], Tianyi Xu[2], Peng Xu[1] and Shihua Wu[1, 2]*

[1] Joint Research Centre for Engineering Biology, Zhejiang University-University of Edinburgh Institute, Zhejiang University, Haining 314400, China
[2] Research Center of Siyuan Natural Pharmacy and Biotoxicology, College of Life Sciences, Zhejiang University, Hangzhou, Zhejiang Province 310058, China
* Correspondence: Drwushihua@zju.edu.cn, Shihuawu@intl.zju.edu.cn; Tel.: +86-13968131541

Legends for Supplementary figures

Figure S1. The chemical structures of major tanshinones isolated from *Salvia miltiorrhiza* rhizome.
Figure S2. The effects of DS-1 on HeLa cell adhesion on cell culture plates against trypsin digestion. (**A**) Hela cells were seeded at 80000 per well in 6-well plates for 12 h, then treated with 0, 10.0, 15.0, 20.0 μM DS-1 for 24 h. After that, cells were washed by PBS solution and digested by 400 μL 0.25% trypsin. During the digestion, cellular morphology was recorded by inverted microscope with camera every 30 seconds. The digestion time was shown in Figure (**B**).
Figure S3. The effects of DS-1 on NRK-49F cell adhesion on cell culture plates against trypsin digestion. (**A**) NRK-49F cells were seeded at 80000 per well in 6-well plate for 12 h, then treated with 0, 6.25, 12.5 μM DS-1 for 24 h. After that, cells were washed by PBS solution and digested by 400 μL 0.25% trypsin. During the digestion, cellular morphology was recorded by inverted microscope every 30 seconds. The digestion time was shown in Figure (**B**).
Figure S4. CD44 knockdown by siRNA. (**A**) U-2 OS cells were cultured in the 6-well plate, then CD44 was knocked down by siRNA for 24 h and 36 h. The relative expression of CD44 was detected by Western Blot and shown in Figure (**B**).

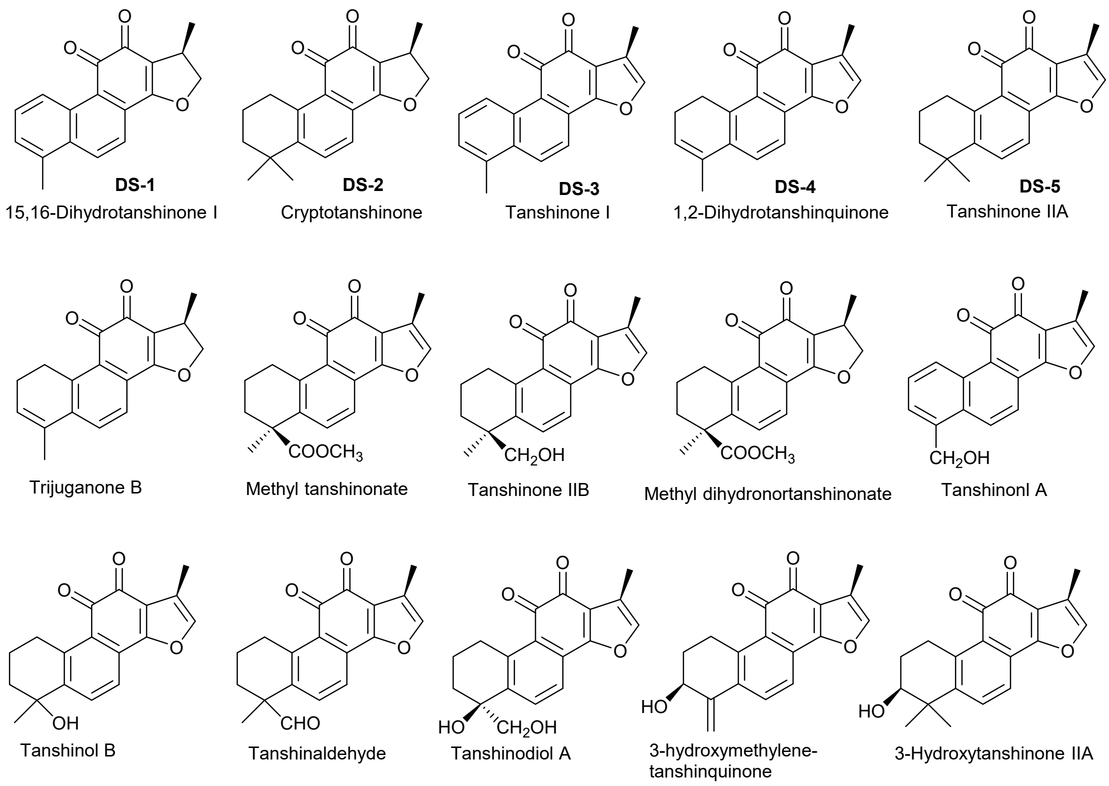

Figure S1. The chemical structures of major tanshinones isolated from *Salvia miltiorrhiza* rhizome.

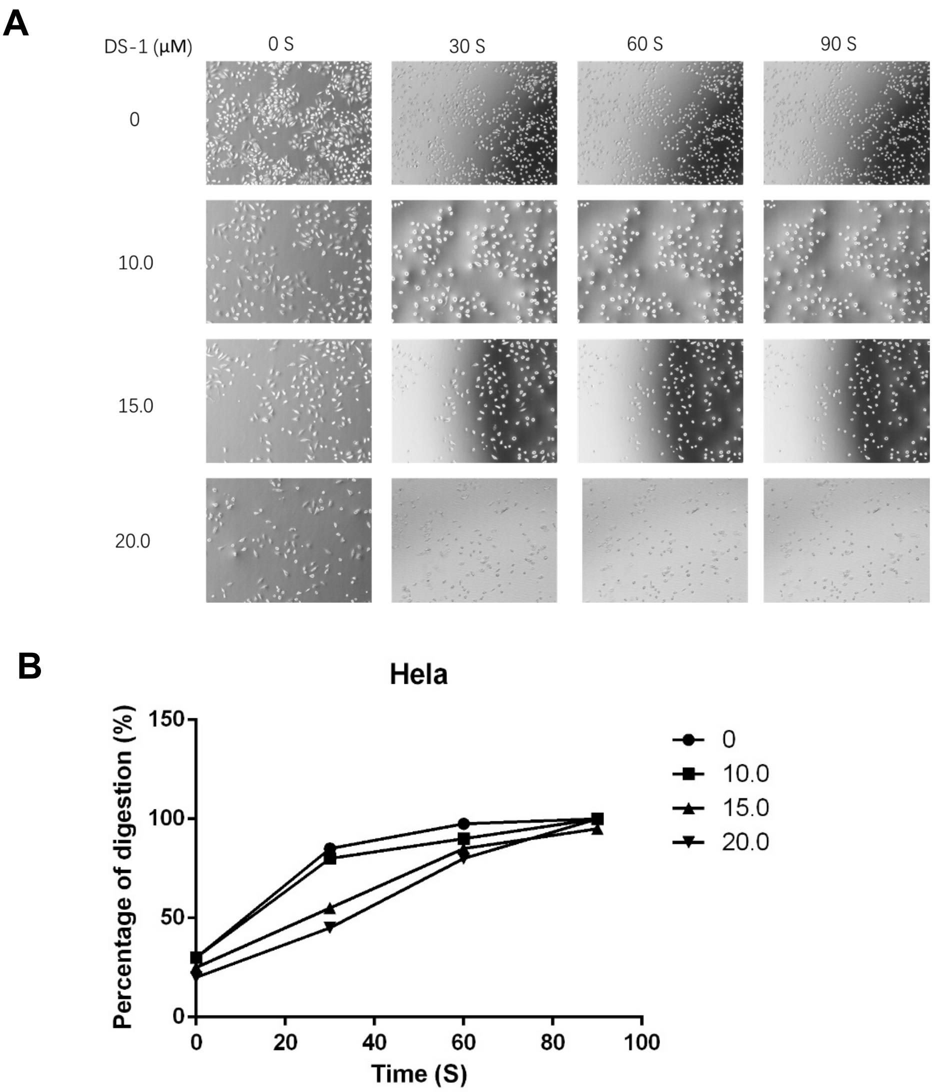

Figure S2. The effects of DS-1 on HeLa cell adhesion on cell culture plates against trypsin digestion. (A) Hela cells were seeded at 80000 per well in 6-well plates for 12 h, then treated with 0, 10.0, 15.0, 20.0 μM DS-1 for 24 h. After that, cells were washed by PBS solution and digested by 400 μL 0.25% trypsin. During the digestion, cellular morphology was recorded by inverted microscope with camera every 30 seconds. The digestion time was shown in Figure (**B**).

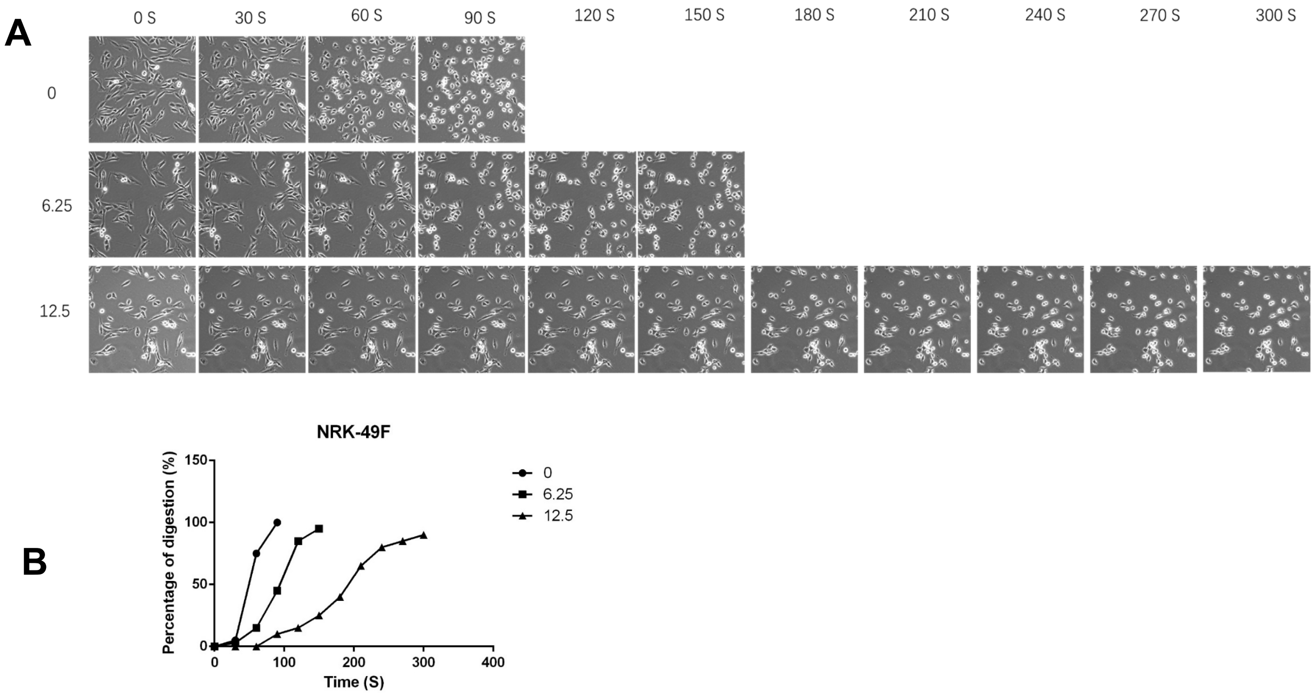

Figure S3. The effects of DS-1 on NRK-49F cell adhesion on cell culture plates against trypsin digestion. (**A**) NRK-49F cells were seeded at 80000 per well in 6-well plate for 12 h, then treated with 0, 6.25, 12.5 μM DS-1 for 24 h. After that, cells were washed by PBS solution and digested by 400 μL 0.25% trypsin. During the digestion, cellular morphology was recorded by inverted microscope every 30 seconds. The digestion time was shown in Figure (**B**).

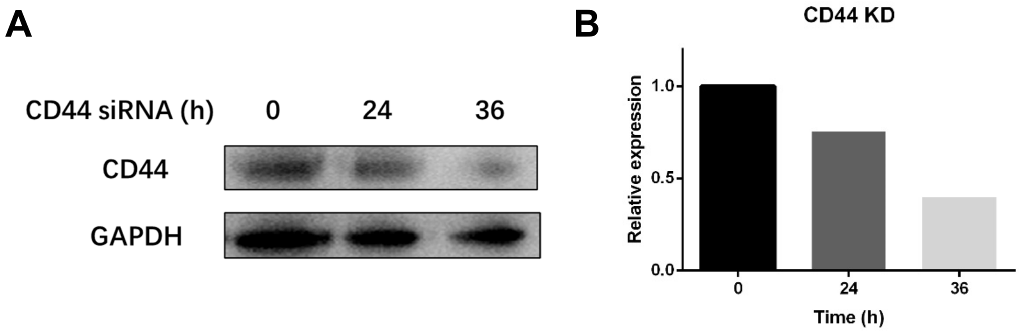

Figure S4. CD44 knockdown by siRNA. (**A**) U-2 OS cells were cultured in the 6-well plate, then CD44 was knocked down by siRNA for 24 h and 36 h. The relative expression of CD44 was detected by Western Blot and shown in Figure (**B**).
